# Complete Genome Sequence of *Moraxella catarrhalis* Strain CCRI-195ME, Isolated from the Middle Ear

**DOI:** 10.1128/genomeA.00384-17

**Published:** 2017-05-25

**Authors:** Aimee Tan, Luke V. Blakeway, Lauren O. Bakaletz, Matthew Boitano, Tyson A. Clark, Jonas Korlach, Michael P. Jennings, Ian R. Peak, Kate L. Seib

**Affiliations:** a Institute for Glycomics, Griffith University, Gold Coast, Australia; b Center for Microbial Pathogenesis, Research Institute at Nationwide Children’s Hospital, Ohio State University College of Medicine, Columbus, Ohio, USA; c Pacific Biosciences, Menlo Park, California, USA

## Abstract

*Moraxella catarrhalis* is an important bacterial pathogen that causes otitis media and exacerbations of chronic obstructive pulmonary disease. Here, we report the complete genome sequence of *M. catarrhalis* strain CCRI-195ME, which contains the phase-variable epigenetic regulator ModM3.

## GENOME ANNOUNCEMENT

*Moraxella catarrhalis* is a human-specific pathogen that causes otitis media and exacerbations of chronic obstructive pulmonary disease (1, 2), but it can also be carried asymptomatically in the nasopharynx. The reason for differences in clinical presentation remains unclear, as genome analysis of *M. catarrhalis* isolates suggests a highly conserved genome with virulence determinants present in strains of both pathogenic and nonpathogenic origins (3, 4).

One potential explanation for the differences in pathology is differential regulation. Previous studies show that *M. catarrhalis* strains have a phase-variable DNA methyltransferase, ModM, that can alter the expression of a phase-variable regulon, known as a phasevarion (5, 6). Three alleles of ModM have been identified that have distinct DNA recognition domains and are hypothesized to alter different phasevarions. The *modM3* allele is more frequently associated with strains isolated from the middle ear during otitis media than the nasopharynx during asymptomatic carriage (5). We report here the complete genome sequence of strain CCRI-195ME, an *M. catarrhalis* strain isolated from the middle ear of a 16-month-old child prone to otitis media (approved by Nationwide Children’s Hospital Institutional Review Board). CCRI-195ME contains the *modM3* allele (MC195_06605), and this is the first closed genome of an *M. catarrhalis* strain with this allele.

Sequenced DNA was extracted from *M. catarrhalis* CCRI-195ME and grown on GC agar overnight at 37°C with 5% CO_2_, using the GenElute bacterial genomic DNA kit (Sigma-Aldrich), per the manufacturer’s instructions. The DNA was sequenced with the PacBio RSII platform using two SMRT cells and 180-min acquisition (Pacific Biosciences). Genome sequences were assembled *de novo* using the hierarchical genome assembly process (HGAP) (7). Consensus sequences generated using Quiver were submitted to NCBI for annotation with the Prokaryotic Genome Annotation Pipeline (PGAP), and annotated sequences were submitted to GenBank.

The CCRI-195ME genome resolved into a complete 1,954,607-bp chromosome (41.6% G+C) and a 39,779-bp plasmid (39.5% G+C). The genome consists of 1,782 predicted coding genes and 66 RNA genes (16 rRNAs and 50 tRNAs). CCRI-195ME belongs to 16S rRNA subtype I, as described in (8), and contains many of the known *M. catarrhalis* virulence determinants and vaccine candidates, including MID/Hag, CopB, UspA2H, and the transferrin-binding proteins TbpA and TbpB (9). The CCRI-195ME plasmid contains 44 predicted open reading frames. Overall, the plasmid does not show similarity to any plasmids in the NCBI databases. However, it does contain a number of putative homologues to transfer operon (Tra locus) components (10), suggesting that the plasmid may be conjugative.

PacBio methylome analysis determined that the ModM3 DNA methylation site 5′-AC^m6^ATC-3′ is present 4,529 times in the genome (4,446 in the chromosome and 83 in the plasmid) and is methylated to 100% when ModM3 is expressed. Collectively these data will aid in the understanding of how ModM3 mediates regulation and will help define the stably expressed immunological target repertoire of this organism. This will provide insight into the pathobiology of *M. catarrhalis* and aid the development of antibacterial strategies and vaccines.

### Accession number(s).

The CCRI-195ME genome has been deposited in GenBank under the accession numbers CP018059 (chromosome) and CP018060 (plasmid).

## References

[1] de Vries SPW, Bootsma HJ, Hays JP, Hermans PWM 2009 Molecular aspects of *Moraxella catarrhalis* pathogenesis. Microbiol Mol Biol Rev 73:389–406. doi:10.1128/MMBR.00007-09. 19721084 PMC2738133

[2] Murphy TF, Parameswaran GI 2009 *Moraxella catarrhalis*, a human respiratory tract pathogen. Clin Infect Dis 49:124–131. doi:10.1086/599375. 19480579

[3] Davie JJ, Earl J, de Vries SP, Ahmed A, Hu FZ, Bootsma HJ, Stol K, Hermans PW, Wadowsky RM, Ehrlich GD, Hays JP, Campagnari AA 2011 Comparative analysis and supragenome modeling of twelve *Moraxella catarrhalis* clinical isolates. BMC Genomics 12:70. doi:10.1186/1471-2164-12-70. 21269504 PMC3045334

[4] Earl JP, de Vries SPW, Ahmed A, Powell E, Schultz MP, Hermans PWM, Hill DJ, Zhou Z, Constantinidou CI, Hu FZ, Bootsma HJ, Ehrlich GD 2016 Comparative genomic analyses of the *Moraxella catarrhalis* serosensitive and seroresistant lineages demonstrate their independent evolution. Genome Biol Evol 8:955–974. doi:10.1093/gbe/evw039. 26912404 PMC4860680

[5] Blakeway LV, Power PM, Jen FE, Worboys SR, Boitano M, Clark TA, Korlach J, Bakaletz LO, Jennings MP, Peak IR, Seib KL 2014 ModM DNA methyltransferase methylome analysis reveals a potential role for *Moraxella catarrhalis* phasevarions in otitis media. FASEB J 28:5197–5207. doi:10.1096/fj.14-257856. 25183669 10.1096/fj.14-256578

[6] Seib KL, Peak IR, Jennings MP 2002 Phase variable restriction-modification systems in *Moraxella catarrhalis*. FEMS Immunol Med Microbiol 32:159–165. 11821238 10.1111/j.1574-695X.2002.tb00548.x

[7] Chin CS, Alexander DH, Marks P, Klammer AA, Drake J, Heiner C, Clum A, Copeland A, Huddleston J, Eichler EE, Turner SW, Korlach J 2013 Nonhybrid, finished microbial genome assemblies from long-read SMRT sequencing data. Nat Methods 10:563–569. doi:10.1038/nmeth.2474. 23644548

[8] Bootsma HJ, van der Heide HGJ, van de Pas S, Schouls LM, Mooi FR 2000 Analysis of *Moraxella catarrhalis* by DNA typing: evidence for a distinct subpopulation associated with virulence traits. J Infect Dis 181:1376–1387. doi:10.1086/315374. 10762569

[9] Ren D, Pichichero ME 2016 Vaccine targets against *Moraxella catarrhalis*. Expert Opin Ther Targets 20:19–33. doi:10.1517/14728222.2015.1081686. 26565427 PMC4720561

[10] Frost LS, Ippen-Ihler K, Skurray RA 1994 Analysis of the sequence and gene products of the transfer region of the F sex factor. Microbiol Rev 58:162–210. 7915817 10.1128/mr.58.2.162-210.1994 PMC372961

